# Endothelial fatty liver binding protein 4: a new targetable mediator in hepatocellular carcinoma related to metabolic syndrome

**DOI:** 10.1038/s41388-018-0597-1

**Published:** 2018-12-21

**Authors:** Samira Laouirem, Aurélie Sannier, Emma Norkowski, François Cauchy, Sabrina Doblas, Pierre Emmanuel Rautou, Miguel Albuquerque, Philippe Garteiser, Laura Sognigbé, Jerôme Raffenne, Bernard E. van Beers, Olivier Soubrane, Pierre Bedossa, Jerôme Cros, Valérie Paradis

**Affiliations:** 1 INSERM UMR 1149, Centre de Recherche sur l’Inflammation, Paris, France; 2 Hepatobiliary Surgery Dept., Beaujon Hospital, Clichy, France; 3 Hepatology Dept., Beaujon Hospital, 100 bd General Leclerc, 92110 Clichy, France; 4 Pathology Dept., Beaujon Hospital, Clichy, France

**Keywords:** Cancer microenvironment, Mechanisms of disease

## Abstract

Metabolic syndrome (MS) is becoming the leading risk factor for hepatocellular carcinoma (HCC). HCC development related to MS may occur in advanced or non-advanced liver fibrosis, suggesting specific molecular pathways. Among these pathways, basal inflammatory state and adipokines production are involved. The aim of this study was to evaluate the role of fatty acid-binding protein 4 (FABP4). In this study, we demonstrate the specific overexpression of FABP4 in human HCC samples from patients with MS compared to other risk factors for chronic liver disease with FABP4 expression restricted to peritumoral endothelial cells. In vitro, glucose, insulin, VEGFA and hypoxia upregulated endothelial FABP4, which was reversed by metformin through mTOR pathway inhibition. FABP4 exerts oncogenic effects on hepatoma cell lines by upregulating the angiogenesis gene signature and pathways involved in the cell cycle, leading to increased cell proliferation and migration, and downregulating HIF1 pathway; effects were reversed in the presence of a specific FABP4 inhibitor (BMS309403). We showed the role of microvesicles as FABP4 vectors between endothelial and tumor cells. In vivo, BMS309403 significantly reduces tumor growth in heterotopic and orthotopic xenografted mice model. In conclusion, this study demonstrates the emerging oncogenic role of liver endothelial cells through FABP4 in HCC related to MS, and highlights new anti-neoplastic mechanism of metformin.

## Introduction

Liver carcinogenesis related to metabolic syndrome (MS) is becoming a major challenge with the increase in overweight patients and type 2 diabetes worldwide [1, 2]. Indeed, hepatocellular carcinoma (HCC) associated with MS is now one of the leading indications for liver transplantation [3, 4]. Like other chronic liver diseases, the development of HCC is a multistep process associated with sequential morphological events [steatosis, steato-hepatitis and fibrosis/cirrhosis] that define the wide spectrum of non-alcoholic fatty liver diseases (NAFLD). However, several groups, including ours, have reported that HCC related to MS occurs more frequently in the absence of underlying cirrhosis than HCC related to either chronic alcohol consumption or hepatitis C virus infection [5, 6]. This finding suggests that MS alone might favor the development of HCC. This hypothesis is further supported by the fact that obesity and type 2 diabetes, through specific mediators, are independently associated with the development of HCC [7–9].

Obesity is characterized by chronic low-grade systemic inflammation, in which the expansion of adipose tissue may play a key role through the production of pro-inflammatory cytokines and adipokines [10]. Among these proteins, high leptin levels are associated with the development and progression of various malignancies, including in the liver, while adiponectin is reduced in obesity and inversely correlated with cancer [11, 12]. More recently, additional adipokines regulated by obesity have been isolated in the secretome from adipocytes, including fatty acid-binding protein 4 (FABP4), also known as adipocyte-type fatty acid binding protein (A-FABP). FABP4 is a lipid chaperone protein from a family of 14 to 15 kDa proteins that bind with high affinity to hydrophobic ligands, including saturated and unsaturated long-chain fatty acids [13]. FABP4 has been shown to play a crucial role in insulin resistance, type 2 diabetes, and atherosclerosis [14–17]. Indeed, *Fabp4*^*−/−*^ mice are protected from obesity-induced insulin resistance and hyperglycemia [18]. In addition to the increased FABP4 circulating levels observed in experimental models of obesity, a positive correlation between FABP4 levels, obesity and type 2 diabetes has been reported in several cohorts of patients [19, 20]. Moreover, circulating FABP4 levels are increased in patients with NAFLD and correlated with liver inflammation and fibrosis [21, 22].

The involvement of FABP4 in human carcinogenesis is a subject of debate, and results differ depending on tumor type [23–25]. Nevertheless, recent studies showed that adipocytes surrounding tumor cells provide energy by supplying fatty acids to cancer cells through FABP4 [25, 26]. Although FABP4 overexpression has been demonstrated in MS and NAFLD, its influence on human liver carcinogenesis has not been investigated. Indeed, recent data reported increased FABP4 circulating levels in patients with HCC compared to controls and in liver tissue from a chemically induced HCC model submitted to a high fat diet (HFD) [27]. In this study, we demonstrated the specific endothelial overexpression of FABP4 in human HCC related to MS and its pro-oncogenic effects, supporting FABP4 as an attractive adipokine target in HCC patients. Leveraging the availability of liver specimens from patients with MS-related HCC receiving metformin, we discovered a new molecular pathway of the anti-tumoral effect of metformin through FABP4 downregulation.

## Results

### FABP4 expression in liver carcinogenesis related to MS is regulated by metformin

**Fig. 1:**
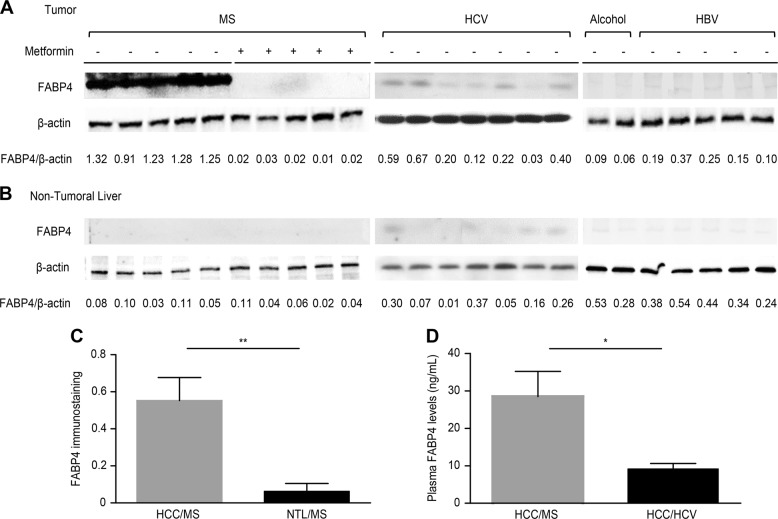
FABP4 is overexpressed in HCC from patients with MS-related chronic liver disease. Representative western blot analysis of FABP4 expression in HCC (**a**) and non-tumoral liver (**b**) from MS (with and without metformin treatment) and non-MS patients (*HCV* Hepatitis C Virus, *HBV* Hepatitis B Virus). **c** Quantitative analysis of FABP4 immunostaining in HCC and paired non-tumoral liver samples from MS patients. **d** Circulating FABP4 levels quantified by ELISA in HCC patients with MS or HCV infection

**Fig. 2:**
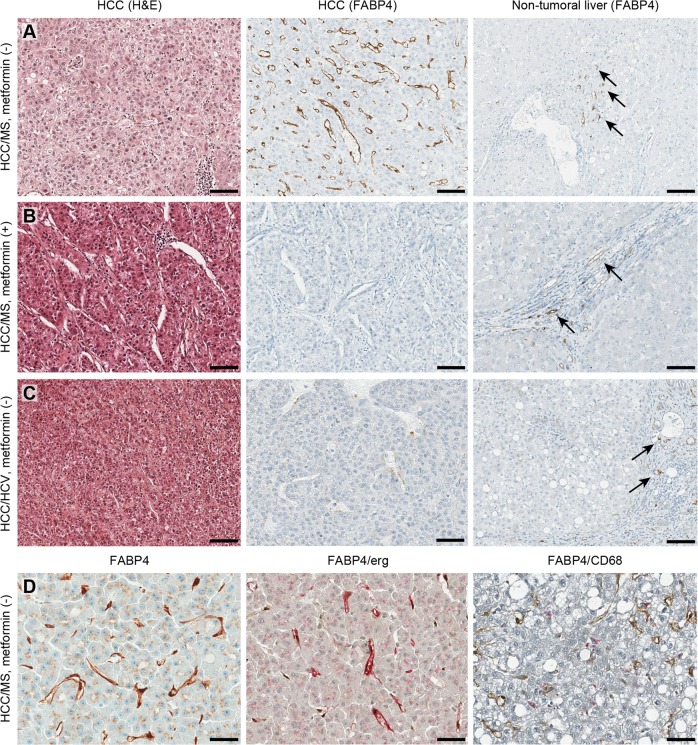
FABP4 is mostly expressed in endothelial peritumoral cells in HCC related to MS. **a** HCC/MS without metformin treatment. **b** HCC/MS with metformin treatment. **c** HCC/HCV. Three panels include hematoxylin & eosin staining of HCC (left), FABP4 immunostaining in HCC (middle) and FABP4 immunostaining in non-tumoral liver (right). Arrows indicate positive vessels in portal tracts and fibrous bands. (Scale bars = 100 μm). **d** FABP4 immunostaining in HCC/MS without metformin. (left) higher magnification showing slight staining was observed in tumoral hepatocytes; (middle) double immunostaining with FABP4 (pink) and ERG (endothelial cells, brown); (right) double immunostaining with FABP4 (brown) and CD68 (macrophages, pink)

Although several studies have reported increased FABP4 expression in liver tissue from patients with insulin resistance, its expression during liver carcinogenesis and the potential regulatory effect of metformin, the most common antidiabetic treatment used in this context, have not been previously studied [28, 29]. FABP4 expression evaluated by western blotting was significantly higher in HCC from patients with MS without metformin compared to MS patients with metformin or other risk factors, including chronic alcohol consumption or HCV and HBV infection (*p* < 0.0001) (Fig. 1a). In all cases, no significant FABP4 expression was observed in non-tumoral livers (Fig. 1b). Immunohistochemistry performed in 21 additional cases from patients with MS (median age 69 years, all men) confirmed, by a computer-assisted quantitative analysis using a dedicated algorithm, the FABP4 overexpression in HCC compared to non-tumoral livers (0.55 *vs*. 0.06, *p* = 0.001) (Fig. 1c). Serum FABP4 levels were also significantly increased in HCC patients with MS compared to HCV (28.5 vs. 9.1 ng/ml, *p* = 0.02) (Fig. 1d). Representative cases of FABP4 immunostaining in HCC illustrated in Fig. 2 shows that FABP4 was predominantly found in sinusoidal cells of HCC/MS without metformin, while few vascular channels within the portal tracts and fibrous bands were labelled in non-tumoral livers (panel a). No sinusoidal staining was observed in HCC from patients with MS with metformin (panel b**)** or from patients with HCV (panel c). To identify the type of FABP4 positive sinusoidal cells, double immunostaining was performed, and showed that endothelial cells (ERG-positive cells) were the main cell type expressing FABP4 in HCC while few macrophages (CD68-positive cells) were identified (panel d). At higher magnification, a slight staining was observed in tumoral hepatocytes (panel d). Increased mRNA *FABP4* levels were observed in HCC/MS compared to HCC/HCV (x2.6), but the difference did not reach significance (data not shown).

### FABP4 expression is regulated in human endothelial cells

**Fig. 3:**
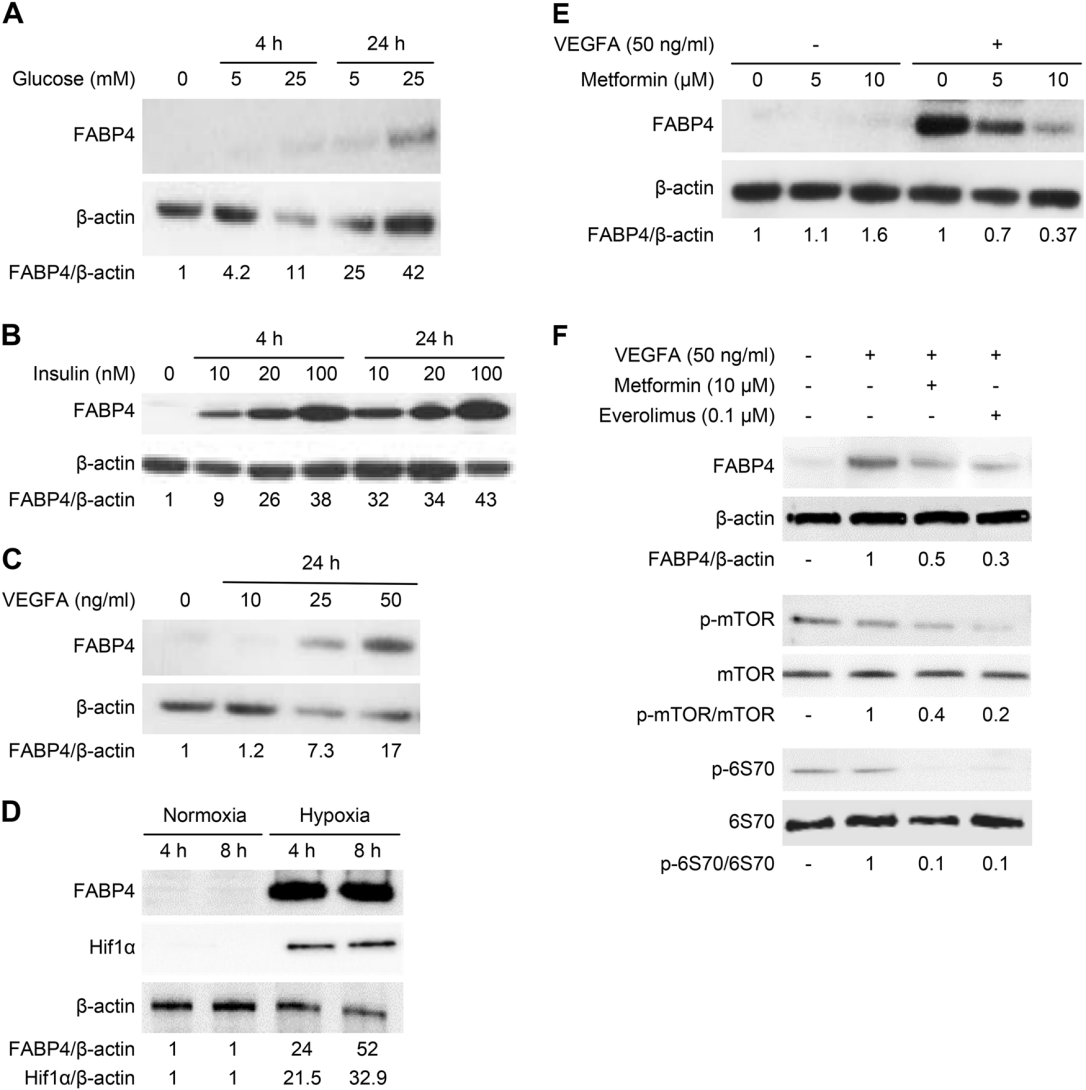
FABP4 is regulated in endothelial cells (HUVEC). FABP4 expression is induced upon stimulation with glucose (**a**), insulin (**b**), VEGFA (**c**) for 4 or 24 h, and hypoxia for 4 and 8 h (**d**). **e** Metformin (5 and 10 µM for 24 h) decreased FABP4 expression in HUVEC stimulated by VEGFA (50 ng/mL for 24 h). **f** Expression of FABP4 and downstream effectors of the mTOR signaling pathway (p-mTOR and p-p70S6K) are decreased in HUVEC stimulated by VEGFA in the presence of metformin (10 μM for 24 h) or everolimus (0.1 μM for 24 h). The ratios FABP4/β-actin, HIF1α/β-actin, p-mTOR/mTOR and p- p70S6K/p70S6K are indicated. The results are expressed as the mean ± SD from three independent experiments

As FABP4 expression was primarily expressed by endothelial cells in HCC/MS, we studied its regulation in HUVEC. While HUVECs did not express FABP4 under basal conditions, a significant increase in FABP4 protein was observed in the presence of glucose (Fig. 3a), and growth factors such as insulin (Fig. 3b) and VEGFA (Fig. 3c) in a dose-dependent fashion at 4 and 24 h. Hypoxia, a common feature of HCC, also induced a dramatic FABP4 expression in parallel to HIF1α expression (Fig. 3d). *FABP4* mRNA levels were also increased in HUVEC incubated in the presence of glucose, insulin, and VEGFA (Supplementary Fig. 1a–c). As the MS is a low-grade inflammatory state associated with elevated TNFα and IL-6 levels, we investigated their effect on FABP4 expression in HUVEC. While there was a concentration-dependent increase in FABP4 protein in the presence of TNFα for 24 h, no effect was observed in the presence of IL-6 (Supplementary Fig. 2).

Based on the strong FABP4 downregulation observed in HCC from MS patients treated with metformin, we evaluated the effects of metformin on endothelial FABP4 regulation. Metformin significantly decreased VEGFA-induced FABP4 expression in a concentration-dependent manner (5 or 10 μM) (Fig. 3e). *FABP4* mRNA level was also significantly decreased by metformin (10 μM) in HUVEC stimulated by VEGFA (*p* = 0.009) (Supplementary Fig. 1d). Since increasing evidence supports the anti-tumoral activity of metformin mainly through mTOR pathway downregulation, we investigated downstream effectors of this pathway [30]. p-mTOR/mTOR and p-p70S6K/p70S6K ratios were significantly decreased in stimulated HUVEC during metformin treatment (Fig. 3f). Similar results were obtained with everolimus (0.1 μM), an mTORC1 complex inhibitor, confirming involvement of the mTOR signaling pathway in FABP4 regulation in HUVEC (Fig. 3f).

### Pro-oncogenic effects of exogenous FABP4 (eFABP4) in hepatoma cell lines

**Fig. 4:**
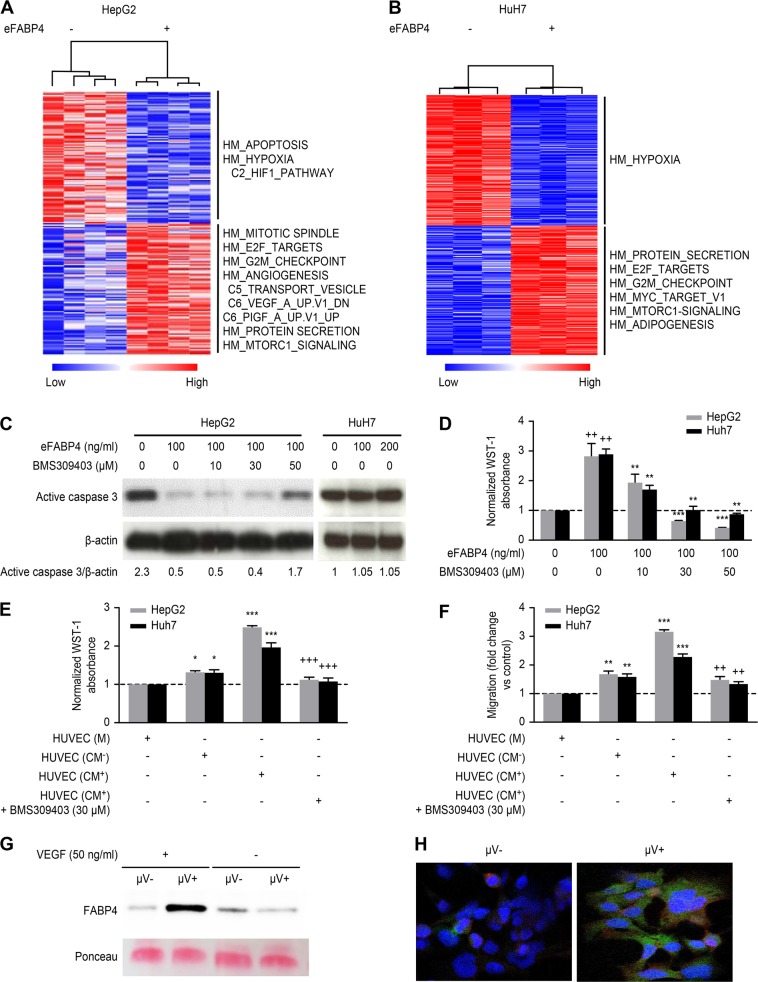
FABP4 pro-oncogenic effects in hepatoma cell lines. Unsupervised hierarchical clustering using the top 300 up (red) and down (blue) regulated genes from microarray data of HepG2 (**a**) and HuH7 cells (**b**) untreated or treated by exogenous FABP4 (eFABP4). Enriched pathways in either condition, derived from the Gene Set Enrichment Analysis stand on the right side. **c** eFABP4 reduces active caspase 3 expression in HepG2 cells (effect is reversed in presence of BSM309403) while no effect is observed in HuH7 cells. **d** Effect on cell proliferation of FABP4 inhibitor (BMS309403) at increasing concentrations on stimulated HepG2 and HuH7 by FABP4 [eFABP4 vs. control (++) *p* < 0.01; BMS309403 vs. eFABP4 **p < 0.01; ***p < 0.001]. Effect of medium (M) and VEGFA stimulated HUVEC CM (CM^+^) or not (CM^-^) on HepG2 and HuH7 proliferation (**e**) and migration (**f**) in the presence or absence of BMS309403 [CM vs. M *p < 0.05; ***p < 0.001; CM with BMS309403 vs. CM without BMS309403 ^++^*p* < 0.01; ^+++^*p* < 0.001]. The results are expressed as the mean ± SD from 3 independent experiments. **g** Immunoblot of FABP4 in CM^+^ or CM^−^ containing (μV+) or not (μV−) microvesicles. **h** Double immunofluorescence of HepG2 cells incubated with CM^**+**^ containing (μV+) or not (μV-) microvesicles (FABP4: green; red: β-catenin; DAPI: blue)

FABP4 has been shown to play a role in cancer cell growth and metastasis in several cancers, including ovarian, breast and oral squamous carcinomas [25, 31–33]. We did not observe FABP4 expression in hepatoma cell lines (HepG2, SKHep1 and HuH7) (Supplementary Fig. 3). We performed gene expression to identify the molecular pathways that are potentially deregulated in HepG2 and HuH7 cells during eFABP4 stimulation to determine whether FABP4 affects tumor cells. Gene set enrichment analysis of whole transcriptome analysis data showed that eFABP4 stimulation led to upregulation of common pathways, including the angiogenesis signature and cell cycle regulation (G2/M checkpoint and mitotic spindle) together with a downregulated HIF1 pathway (Fig. 4a, b). In contrast, while eFABP4 reduced the apoptotic pathway in HepG2 cells, no effect was observed in HuH7 cells. We confirmed this differential effect by quantifying active caspase-3 by western blotting and showed that active caspase 3 reduction was reversed by increasing concentrations (10 to 50 μM) of a specific FABP4 inhibitor (BMS309403) (Fig. 4c).

Based on the results of gene expression, we confirmed the effects of eFABP4 (100 ng/mL for 24 h) on cell viability and proliferation in vitro using HepG2 and HuH7 cells, and this effect was also significantly reversed in the presence of BMS309403 (Fig. 4d, e). To understand the crosstalk between endothelial and hepatoma cells, we evaluated HepG2 and HuH7 cell proliferation and migration with HUVEC conditioned medium (CM) under different conditions. VEGFA-stimulated HUVEC-CM (CM^+^) increased cell proliferation and migration compared to non-stimulated HUVEC-CM (CM^−^). Co-incubation with 30 μM FABP4 inhibitor reversed both effects (Fig. 4e, f). Taken together, these data suggest that VEGFA increases FABP4 expression in HUVEC but also enhances FABP4 release in their CM, thus stimulating cell proliferation and migration. Next, we performed FABP4 western blotting on microvesicles-depleted (μV−**)** or non-depleted (μV+**)** HUVEC-CM and observed that VEGFA increased FABP4 levels in the microvesicles fraction of HUVEC-CM^+^ (Fig. 4g). Next, we incubated HepG2 cells with fluorescently labeled microvesicles obtained from HUVEC-CM^+^ and identified microvesicles bound to the HepG2 cell plasma membrane and within the cytoplasm by confocal immunofluorescence (Fig. 4h).

### FABP4 regulation in liver sinusoidal endothelial cells (LSEC)

**Fig. 5:**
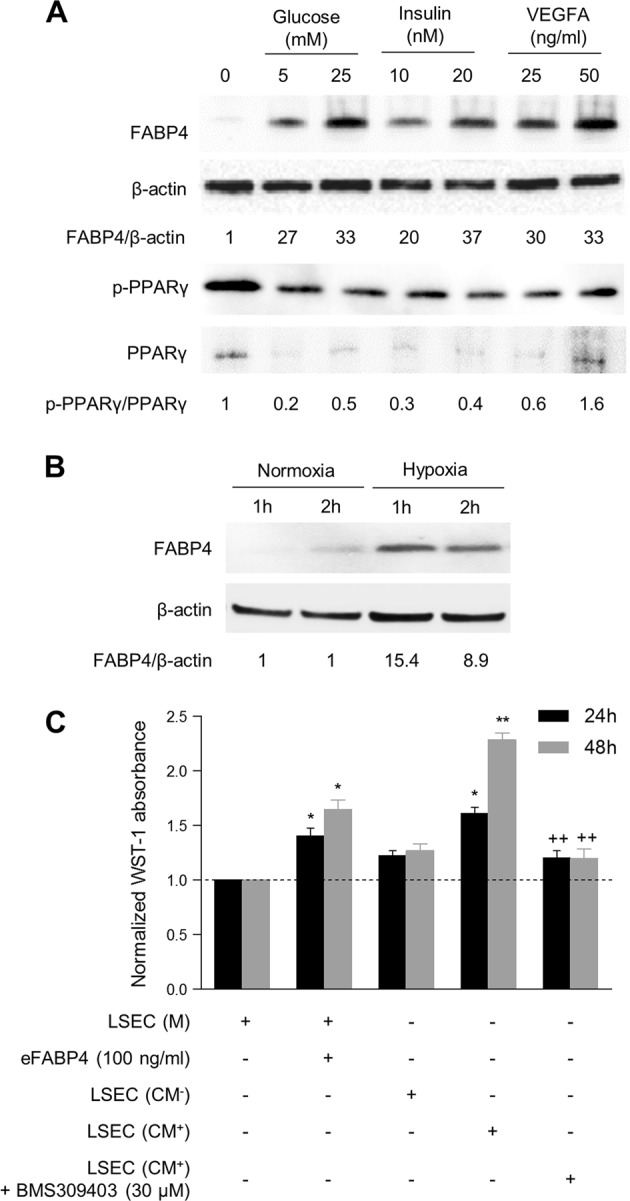
FABP4 is regulated in primary liver sinusoidal endothelial cells (LSEC) and exerts pro-oncogenic effects on HepG2 cells. **a** FABP4, PPARγ, and p-PPARγ expression in LSEC upon stimulation with glucose (5 and 25 mM), insulin (10 and 20 nM) and VEGFA (25 and 50 ng/ml) for 24 h. **b** FABP4 expression in LSEC subjected to hypoxia for 1 and 2 h. **c** Effect of medium (M) and conditioned medium of LSEC stimulated (CM^+^) or not (CM^−^) by VEGFA on HepG2 proliferation in presence or absence of BMS309403 [eFABP4 or CM vs. control *p < 0.05; **p < 0.01; CM with BMS309403 vs. CM without BMS309403 ^++^*p* < 0.01]. The results are expressed as the mean ± SD from three independent experiments

To increase the relevance of our data obtained from HUVEC in human liver carcinogenesis, we studied FABP4 regulation in LSEC. While LSEC did not express FABP4 under basal conditions, a significant increase in FABP4 protein was observed in the presence of glucose (5 and 25 mM), insulin (10 and 20 nM), and VEGFA (25 and 50 ng/ml) for 24 h (Fig. 5a). Given the interrelationship between FABP4 and PPARγ, where PPARγ induces FABP4 which in turn negatively regulates PPARγ in adipocytes and macrophages, we evaluated the effect of FABP4 upregulation on PPARγ expression and its phosphorylated form [34]. We demonstrated that whatever the stimulus, FABP4 increase was associated with PPARγ down regulation and decrease in the ratio p-PPARγ/PPARγ in most of the conditions (Fig. 5a). Hypoxia (for 1 and 2 h) also induced significant FABP4 expression (Fig. 5b). Similar to HUVEC, VEGFA-stimulated LSEC-CM increased HepG2 cell viability and proliferation, and co-incubation with 30 μM of FABP4 inhibitor reversed this effect (Fig. 5c).

### FABP4 regulation in hepatoma cell lines

While no basal expression of FABP4 was observed in hepatoma cell lines (Supplementary Fig. 3a), we evaluated the effect of hypoxia (a common feature of malignancy) on FABP4 expression in HepG2 and HuH7 cells. Hypoxia for 24 h induced a de novo FABP4 expression in both cell lines (Supplementary Fig. 3b); no induction was observed at 4h (data not shown). As MS is associated with free fatty acids (FFA) increase, we also evaluated the impact of FFA on FABP4 expression. Both palmitic acid (PA) and oleic acid (OA) were able to induce FABP4 expression in HepG2 and HuH7 cell lines, with a greater increase with OA and combination of PA and OA compared to PA (Supplementary Fig. 3c).

### FABP4 inhibition decreases tumor growth in vivo

**Fig. 6:**
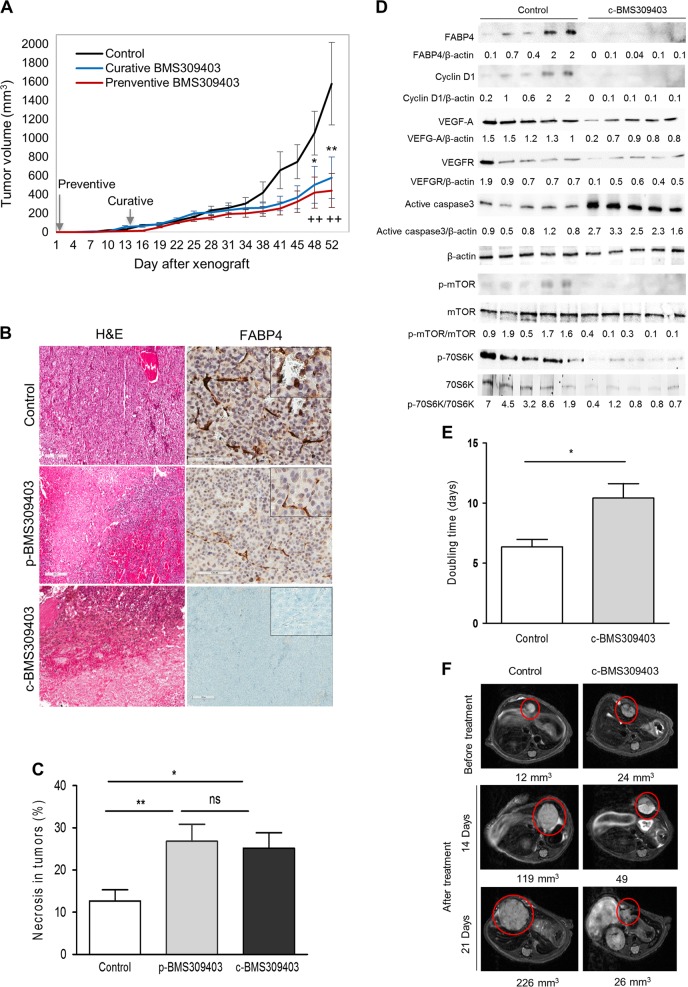
FABP4 inhibition decreases tumor growth in vivo (Heterotopic model (a-d), Orthotopic model (e-f). **a** Tumor volume (mm^3^) was measured twice a week in the 3 groups of mice. The figure indicates the tumor burden volume versus time since xenograft (in days). Data are expressed as mean values and standard deviation for each point. **b** Morphological aspect (left, Hematoxylin & Eosin) and FABP4 immunostaining (right) of representative tumors from the 3 different groups [control (top), p-BMS309403 (middle) and c-BMS309403 (below)]. **c** Percentage of tumor necrosis was assessed by hematoxylin & eosin (HE) staining for each group of mice [BMS309403 vs. control *p < 0.05; **p < 0.01]. **d** Expression of FABP4, Cyclin D1, VEGFA, VEGFR, caspase 3, mTOR, phospho-mTOR, 70S6K and phospho-70S6K in tumors from control and treated (curative BMS309403 treatment) mice (*n* = 5 each). **e** Tumor doubling time of intrahepatic tumors (days) was calculated using MR imaging in control (*n* = 5) and treated mice (*n* = 6). **f** T2-weighted morphological MR images obtained in a control (left) and a treated mouse (right) before treatment, at 2 weeks and 3 weeks after treatment initiation. The intrahepatic tumors are circled in red and the total volume of tumors is indicated (mm^3^)

To assess the potential anti-tumoral effect of FABP4 in vivo, we measured tumor growth in two experimental HFD mice model xenografted (heterotopic and orthotopic) with HepG2 cells (Fig. 6a). Significant weight gain was observed in mice submitted to HFD between the 12^th^ (starting HFD, 18.9 ± 0.7 g) and the 28^th^ week (time to xenograft, 30.3 ± 5.4 g, *p* < 0.01) or the 34^th^ week (euthanasia, 27.0 ± 2.7 g, *p* < 0.01) whatever the groups (preventive or curative treatment or controls). At euthanasia, serum glucose levels were not significantly different between the groups (controls: 95 ± 18 mg/dL; preventive: 92.7 ± 27.1 mg/dL and curative: 66.8 ± 30.7 mg/dL) (data not shown).

Ten days after HepG2 cell injections in 24 mice (2 sites/mouse), 36 subcutaneous tumors were observed and measured according to the protocol (10/16 in preventive and 13/16 in curative or control groups, ns). Total tumor volume per mouse was significantly lower in xenografted HFD mice treated with BMS309403 at 48 and 52 days. At 52 days, tumor volumes were lower both in the preventive (442 ± 195 mm^3^, *p* < 0.01) and curative (579 ± 204 mm^3^, *p* < 0.01) groups compared to controls receiving PBS (1579 ± 403 mm^3^) (Fig. 6a). To evaluate the efficacy of BMS309403 on FABP4 inhibition, circulating FABP4 levels were measured by ELISA at euthanasia. Mean levels were significantly decreased in treated groups (preventive vs. control, *p* = 0.001 curative vs. control, *p* = 0.0006) (data not shown). At euthanasia, tumors were collected and histological analysis revealed a significant increase in tumor necrosis in mice receiving BMS309403 (preventive 26.8%, *p* = 0.008; curative 24.3%, *p* = 0.01) compared to controls (12.7%, *p* = 0.01) (Figs. 6b, c). In addition, FABP4 tumor endothelial cell immunostaining was significantly lower in mice treated with BMS309403 both in the preventive group (11.3%, ranges 8.6–22.2, *p* = 0.02) and in the curative group (0.1%, ranges 0.09–4.5, *p* = 0.007) than in controls (45.9%, ranges 44.6–77.6) (Fig. 6b). In vivo effects of FABP4 downregulation were confirmed by western blotting in HCC tumors (Fig. 6d). Curative treatment with BMS309403 significantly decreased tumor FABP4 (*p* = 0.04), cyclin D1 (*p* = 0.02), VEGFA (*p* = 0.002) and VEGFR (*p* = 0.001) expression compared to the control group, while active caspase 3 was induced (*p* = 0.008) in parallel with mTOR pathway downregulation. To increase clinical relevance, we evaluated the efficacy of BMS309403 in an orthotopic xenografted model using HFD mice. Liver tumor engraftment was obtained in 54% of mice (14/26) within 14 days for 12 of them. A total of 11 mice were treated (BMS309403, *n* = 6 and PBS, *n* = 5). Fifteen mice were excluded (extra-hepatic engraftment in 3, absence of tumor in 9 and 3 mice died during protocol). Using MRI follow-up, mean tumor doubling time was significantly higher in mice receiving BMS309403 (10.42 days) compared to the control group (6.36 days, *p* = 0.02) (Fig. 6e). Representative MR imaging of 2 mice treated with BMS309403 or PBS is illustrated in Fig. 6f.

## Discussion

Adipocytes have been shown to promote growth of cancer cells in several human malignancies [35, 36]. The MS is characterized by pathological expansion of adipose tissue which, in turn, contributes to liver pathogenesis and the progression of HCC. In addition to leptin and adiponectin [10–12], we provide further evidence that additional adipokines, and especially FABP4, may play a role in the burden of MS-related HCC in human and experimental models.

Several studies have shown that FABP4 is closely associated with obesity and MS [18, 20, 37]. Moreover, Milner et al. reported increased circulating FABP4 levels in patients with NAFLD which were correlated to insulin resistance and the severity of inflammation and fibrosis in the liver [21]. Although increased *FABP4* mRNA levels have been reported in liver samples from morbidly obese insulin resistant subjects and in patients with fatty liver, FABP4 expression has not been explored in human HCC samples [28, 29]. Interestingly, increased circulating FABP4 levels have been recently reported in HCC patients compared to healthy controls, with only a trend towards NAFLD and HCV patients [27]. We confirmed these data, and moreover we showed a significant increase in FABP4 serum levels in HCC patients with MS compared to HCV patients.

This study reports for the first time FABP4 overexpression in HCC tissues from patients with MS compared to other risk factors. Surprisingly, double immunostaining showed that FABP4 expression in human HCC was mainly restricted to tumor cell-lining endothelial cells. Noteworthy, it has been recently shown a FABP4 overexpression in hepatic stellate cells within HCC [38]. It should be remembered that the FABP family includes different members with unique patterns of tissue expression; FABP4 is mainly restricted to adipocytes and activated macrophages [13, 39]. However, and as our results show, during stimulation of pro-angiogenic factors, including VEGF-A and b-FGF, FABP4 expression has been reported in a subset of endothelial cells in several normal tissues as well as in human tumors [31, 40, 41]. We showed that different stimuli, such as glucose and insulin, which are increased in patients with MS, can upregulate FABP4 in HUVEC and in LSEC, a more relevant cell model, which derives from human liver sinusoidal cells. In addition to VEGFA, we demonstrated that hypoxia, a condition increasing VEGFA expression, also upregulates FABP4 as well as in hepatoma cell lines. Interestingly, FABP4 has been shown to be a HIF1α target gene in an experimental model of liver ischemia/reperfusion injury [42]. We confirmed the endothelial FABP4 tumor expression in an experimental HFD mice xenografted model. As a matter of fact, a predominant hepatocellular FABP4 expression has been reported in the DEN experimental model submitted to HFD [27]. Such differences may be related to the diverse nature of the experimental models used in both studies.

Increasing evidence suggests the key role of metformin in cancer development and prognosis, including HCC, by exerting antineoplastic effects, primarily through mTOR inhibition in tumor cells [43]. In the present study, FABP4 expression was significantly decreased in HCC from patients treated with metformin, with a lack of FABP4 staining in tumor endothelial cells. In addition, the FABP4 downregulation observed in HUVECs by metformin is associated with a decrease in the pmTOR/mTOR and p-p70S6K/p70S6K ratio, indicating the involvement of the mTOR pathway in that process. Interestingly, these findings highlight a new anti-oncogenic mechanism of metformin in HCC through FABP4 downregulation in endothelial cells and support its anti-angiogenic effect observed in human HCC and preclinical models [36]. Interestingly, our data are supported by a recent study showing that endothelial FABP4 targeting by siRNA exerts antiangiogenic and antitumor effects in an ovarian tumor xenograft model [37].

Results on the role of FABP4 in cancer are scarce and conflicting [22–24]. FABP4 was determined to be upregulated in omental metastases compared to primary ovarian cancers and interestingly was restricted to tumor cells located at the interface of omental adipocytes [25]. In addition, a significant decrease in tumor burden was observed in *Fabp4*^−/−^ mice intraperitoneally xenografted with ovarian cancer cell lines. These original data suggest that adipocytes play a key role in tumorigenesis by providing fuel to cancer cells. The endothelial cells in our model could play this supporting role in tumor hepatocytes. Thus, although hepatoma cell lines do not express FABP4, stimulation of hepatoma cell lines with eFABP4 modulated several molecular mechanisms involved in tumor progression, including angiogenesis and the cell cycle. As recently reported [27], we also confirmed in vitro the effect of FABP4 on HCC progression as cell proliferation and migration were increased by incubating hepatoma cell lines with eFABP4, both effects being reversed in the presence of a specific FABP4 inhibitor. However, an anti- apoptotic effect was only observed in HepG2 cells, suggesting that specific signaling networks are involved. Importantly, we demonstrated the in vivo effect of FABP4 by treating xenografted mice subjected to HFD with a specific FABP4 inhibitor, through mTOR pathway inhibition. Moreover, in order to take into account the importance of the tumor environment, we also evaluated the potential of BMS309403 in an orthotopic xenografted HFD mice, and confirmed its significant antineoplastic effect through increasing tumor doubling time in treated mice. Even if BMS309403 is considered as a specific FABP4 inhibitor, it may also affect FABP3 and FABP5 activity at higher concentrations. Although we did not observe any significant changes in FABP3 and FABP5 expression in presence of BMS309403 by western-blot both in vitro (endothelial cells) and in vivo (xenografted tumors) (data not shown), we could not formally exclude a potential effect of this drug on FABP3 and FABP5 activity. At last, as data regarding pharmacokinetic and pharmacodynamic behavior of BMS309403 are lacking so far, further experiments are needed before proposing FABP4 inhibitor in a clinical setting.

**Fig. 7:**
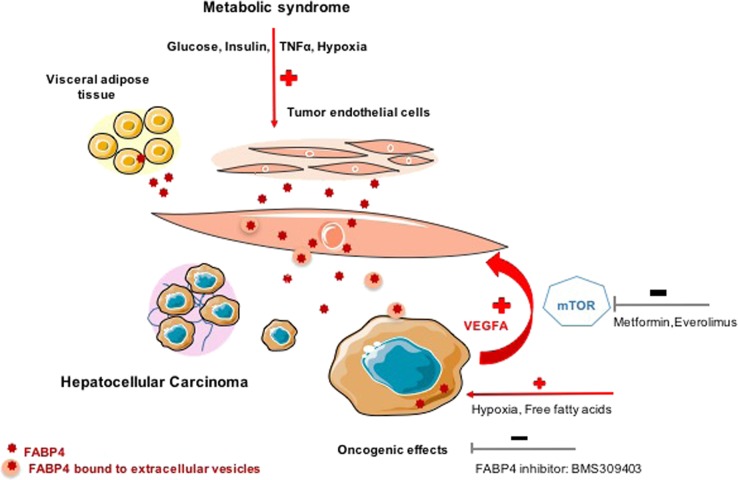
Schematic view recapitulating the involvement of endothelial FABP4 in liver carcinogenesis related to metabolic syndrome

Unlike the study in ovarian cancer, we did not observe FABP4 significant de novo expression in human tumor hepatocytes, suggesting that FABP4 produced by the endothelial cells exerts pro-oncogenic effects on hepatocytes during liver carcinogenesis. Nevertheless, incubation of hepatoma cell lines with free fatty acid treatment led to FABP4 release into the medium [27]. Since no specific FABP4 receptor has been recognized thus far, it has been proposed that Cytokeratin 1 may be used as a receptor in endothelial cells as well as in HepG2 cells [44]. As FABP4 does not contain any secretory signal sequence in its structure, alternative mechanisms, including microvesicles formed by budding from the cell plasma membranes, may be involved, as already described in liver fibrogenesis and portal hypertension [45]. We demonstrated that FABP4 is released by HUVEC and LSEC, and showed in HUVEC that FABP4 is released both free or bound to endothelial-derived microvesicles; the latter can fuse with HepG2 cells to provide them with exogenous FABP4. This non-classical route of FABP4 secretion was recently reported in adipocytes [46]. Figure 7 recapitulates the role of FABP4 in the crosstalk between endothelial and tumoral cells.

In conclusion, our study confirms the oncogenic role of FABP4 in liver carcinogenesis, highlighting the key role of tumor microenvironment via crosstalks between endothelial and tumors cells mainly through microvesicles release from endothelial cells. We also describe a new molecular pathway of the antitumoral effect of metformin through the modulation of FABP4 by endothelial cells. Altogether, our data provide further evidence of the involvement of specific mechanisms, potentially being targeted, in HCC progression in patients with MS.

## Materials and methods

### Human samples

FABP4 expression was studied in resected HCC and paired non-tumoral liver specimens in 25 patients with chronic liver disease, which was related to MS in 10 patients, and unrelated in 15. Among these, 8 had HCV, 2 had alcoholic liver disease, and 5 had HBV. MS was defined according to the current criteria [47]. The non-tumoral liver was evaluated using the Steatosis Activity Fibrosis score in MS patients and the METAVIR score in the other groups [48, 49]. Table 1 summarizes the main clinicopathological features of patients. MS and non-MS patients significantly differ in terms of age (*p* = 0.02), body mass index (BMI, *p* = 0.04), presence of type 2 diabetes (*p* < 0.001) and tumor size (*p* = 0.0005). All patients signed an informed consent form, and the study was approved by the local Ethics Committee (Declaration n° DC-2009-936).

**Table 1:** Clinicopathological characteristics of patients with HCC related to metabolic syndrome (HCC/MS) or not (HCC/non MS)

	HCC/MS			HCC/non MS
	(*n* = 10)	Metformin− (*n* = 5)	Metformin + (*n* = 5)	(*n* = 15)
Median age (range, years)	68 (58-80)	72 (66–78)	62 (58–80)	63 (49–72)
Sex ratio (M/F)	8/2 (80%)	5/0	3/2	13/2 (87%)
BMI (kg/m^2^)	29 (21–43)	28 (21–31)	30 (26–43)	25 (19-37)
Type 2 diabetes	10 (100%)	5	5	0
Metformin treatment	5 (50%)	0	5	0
Median tumor size (range, cm)	7 (2.3-16)	4 (2.3–16)	7 (6.5–13)	3.5 (2–5)
Multiple tumors	2 (20%)	2	0	5 (33.3%)
Well or moderately differentiated HCC	9 (90%)	5	4	14 (93.3%)
Vascular invasion	6 (60%)	2	4	9 (60%)
Satellite nodules	3 (30%)	0	3	2 (13.3%)
Advanced fibrosis in adjacent liver	4 (40%)	3	1	12 (80%)

### Cell culture

FABP4 expression was studied in human umbilical vascular endothelial cells (HUVEC, ATCC, Molsheim, France, < P6) and liver sinusoidal endothelial cells (LSEC, Clinisciences, Nanterre, France) after stimulation with glucose, insulin, VEGFA, TNFα, IL-6, metformin or everolimus for 4 and/or 24 h. FABP4 expression was also studied under hypoxia in LSEC. Concentrations are indicated in Supplemental materials. At the end of the experiment, protein and RNA were collected. FABP4 expression was evaluated in hepatoma cell lines (HepG2, SKHep1 and HuH7) and in 3T3L1 adipocytes at different stages of differentiation (pre, intermediate and mature); the latter was used as the positive control (Supplementary Fig. 3a). Cells were exposed to hypoxia condition with N_2_ at flow rate of 10 l/min for 10 min, and then maintained under hypoxia environment in incubator Chamber (Stem Cell^TM^ Technologies, Vancouver, Canada) for 4 or 24 h. HepG2 and HuH7 cell lines were also incubated in the presence of PA (final concentration 200 μM), or OA (final concentration 200 μM) or in combination for 24 h.

The oncogenic effects of FABP4 were evaluated in HepG2 and HuH7 cells (ATCC®, Manassas, VA, USA). When indicated, 100 or 200 ng/mL exogenous FABP4 (eFABP4, Sigma-Aldrich®, Missouri, USA) and/or 10, 30 and 50 µM BMS309403 (a competitive FABP4 inhibitor which interacts with the fatty-acid binding pocket within the interior of FABP4, R&D systems®, Lille, France) were added to the medium for 24 h [50]. HepG2 cells were also incubated for 24 h with HUVEC or LSEC-conditioned medium (CM) obtained from cells stimulated or not with VEGF (50 ng/mL for 24 and 48 h, respectively), in the presence, when indicated, of 30 µM BMS309403 for 24 h. HepG2 cells incubated in the presence of HUVEC or LSEC medium were used as the control condition. Cell viability and proliferation were assessed using the WST-1 kit (Roche Diagnostics®, Meylan, France). Apoptosis was assessed by anti-active caspase-3 immunoblotting. Cell migration was analyzed using 8 μm transwell inserts (Chemicon International Inc., USA) using the QCM™ 24-Well Colorimetric Cell Migration Assay (Millipore, Billerica, MA, USA). When mentioned, eFABP4 (100 ng/mL) was added to 500 μL of serum free media in the lower chamber for 24 h in the absence or presence of BMS309403 (30 µM for 24 h). All experiments were performed in triplicate.

To test the potential role of microvesicles on the HUVEC-CM incubated with VEGFA (50 ng/mL) for 24 h on HepG2 cells, we removed microvesicles from this medium according to a previously described protocol [45]. CM was centrifuged at 20,500×*g* for 150 min at 4 °C to pellet microvesicles. The supernatant of this centrifugation was filtered on a membrane with 0.10 μm pores to completely remove microvesicles (Minisart filters, Sartorius Stedim Biotech, Goettingen, Germany). This medium was called “μV−” and compared to the CM containing microvesicles (μV+).

### Immunoblotting, immunohistochemistry, double immunofluorescence study, gene expression profiling analysis, and in vivo imaging

See Supplementary Materials.

### Animal experiments

Female BALB/c-nude mice (Harlan Laboratories Milan, Italy) were housed at the animal facility (CRI, Bichat) under specific pathogen-free conditions (22 ± 2 °C, 50% humidity, 12 h light/12 h dark) according to the European Community guidelines and local ethics committee approval (authorization n° C75-18-01). At 12 weeks old, mice were fed a high-fat diet (HFD, 34% crude fat; 60% fat, 17% protein and 23% carbohydrates) (ref # C1090-60%; Altromin®, Lage, Germany) for 22 weeks. After 7 and 22 weeks of HFD, the oral glucose tolerance test was performed to confirm the insulin resistant state as previously described [51]. In the heterotopic in vivo model, HepG2 cells were xenografted after 16 weeks of HFD at 2 subcutaneous sites (4.10^6^/site). A total of 24 nude mice were divided into 3 groups: preventive treatment with a specific FABP4 inhibitor (BMS309403, 45 mg/kg, intraperitoneally twice a week, starting at the time of the xenograft, *n* = 8), curative treatment with BMS309403 (45 mg/kg, twice a week, starting 2 weeks after the xenograft, *n* = 8), and control (receiving PBS at the time of xenograft, *n* = 8) groups. Tumor volume was measured twice a week. At euthanasia (D52 following xenograft), tumors and livers were collected and subjected to histologic examination. Animal blood was collected from the heart and the serum was obtained after 20 min of centrifugation at 2000 g. In the orthotopic in vivo model, after 8 weeks of HFD, HepG2 cells (12.10^6^) were xenografted in the left liver lobe. Treatment was started when tumor volume was ≥10 mm^3^. Six mice received BMS309403 (2 intra-peritoneal injections / week, 45 mg/Kg) for 3 weeks. As a control group, 5 mice received intra-peritoneal injections of PBS (twice a week) for 3 weeks. Tumor progression was followed using MRI and tumor doubling time was calculated (Suppl.file). All mice were sacrificed at the end of treatment.

### Statistical analysis

Continuous variables are given as means (range) and categorical variables as frequencies (percentage). Data were normalized and expressed as fold change. Comparisons between groups of quantitative variables were performed using Student’s *t-*test at a significance level of 0.05 (* or +), 0.01 (** or ++) or 0.001 (*** or +++).

## Supplementary information

supp fig 1

supp fig 2

supp fig 3

Supp material
