# Supplementary figures and images for: Endothelial fatty liver binding protein 4: a new targetable mediator in hepatocellular carcinoma related to metabolic syndrome
Source: Oncogene. 2018 Dec 21;38(16):3033–46. doi: 10.1038/s41388-018-0597-1 (PMC6484689; doi:10.1038/s41388-018-0597-1)

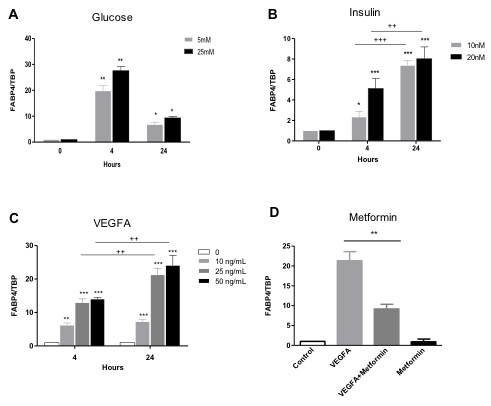

Supplement: Supplementary file 1 — supp fig 1 [file 41388_2018_597_MOESM1_ESM.jpg]

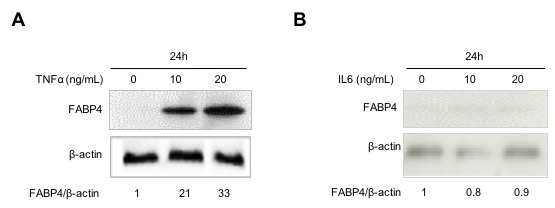

Supplement: Supplementary file 2 — supp fig 2 [file 41388_2018_597_MOESM2_ESM.jpg]

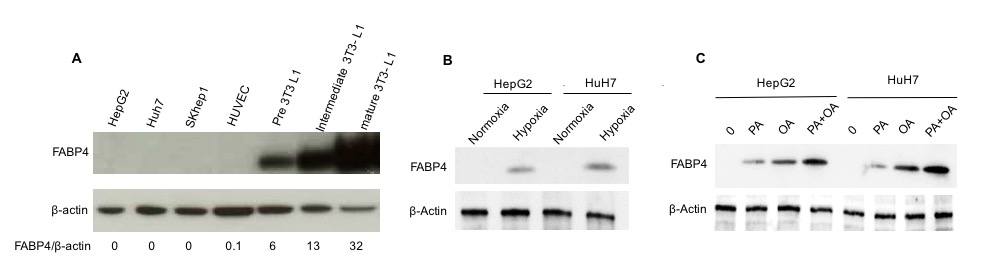

Supplement: Supplementary file 3 — supp fig 3 [file 41388_2018_597_MOESM3_ESM.jpg]
