# Supplementary material for: Endothelial fatty liver binding protein 4: a new targetable mediator in hepatocellular carcinoma related to metabolic syndrome
Source: Oncogene. 2018 Dec 21;38(16):3033–46. doi: 10.1038/s41388-018-0597-1 (PMC6484689; doi:10.1038/s41388-018-0597-1)
Supplement: Supplementary file 4 — Supp material [file 41388_2018_597_MOESM4_ESM.docx]

**Supplementary Materials and Methods**

***Protein Extraction and Western Blotting***

Cultured cells or frozen tissue were homogenized in lysis buffer and extracted by Tissuelyser LT (50 Hz, 15 seconds). Total proteins were determined using the CBX kit (Agro-Bio®, Rennes, France). For immunoblotting, 15-30 µg of protein were resolved via SDS-PAGE (Bio-Rad system®, France) and transferred to PVDF membranes. The membranes were then blocked with 5% bovine serum albumin (Sigma-Aldrich®, Missouri, USA) in Tris-buffered saline with tween-20 (TBST). The membranes were incubated overnight at +4°C with primary antibodies targeting FABP4 (ref # HPA002188, 1:1000, Sigma-Aldrich®, Missouri, USA), caspase 3 (ref # HPA002643, 1:1000, Sigma-Aldrich®, Missouri, USA), HIF1α (ref #ab16066, 1:1000, Abcam, UK), p-mTOR (ref #2976S), mTOR (ref #2983S), p-6S70 and 6S70 (ref #2708S and #9234S, respectively; 1:1000, Cell Signaling Technology, Danvers®, MA, USA), and PPARγ (ref #ab59256, 1:1000, Abcam, UK). Membranes were washed with TBST and incubated with ready-to-use secondary antibody (1:2000, Abcam®, Cambridge, UK). Protein bands were revealed with HRP substrate and results were visualized with the camera Chemic DocTM Touch Imaging System (BioRad®). A primary antibody against β-actin (ref # ab3280, 1:2000, Abcam®, Cambridge, UK) was used as the control and the results were defined by the ratio protein of interest/β-actin.

***Immunohistochemistry***

FABP4 immunostaining was performed with anti-FABP4 polyclonal antibody (ref # HPA002188, 1:400, Sigma-Aldrich®, Missouri, USA) using formalin-fixed paraffin-embedded sections of HCC and non-tumoral livers. Immunostained slides were digitized (Scanscope AT turbo®, Leica), and FABP4 immunostaining was quantified using a dedicated algorithm (quantification pixel positive, Indica labs). Double-staining immunohistochemistry was performed using an anti-FABP4 antibody associated with an anti-CD31 monoclonal mouse antibody (ref # M082329-2, 1:200, Dako, Les Ulis, France), anti-ERG monoclonal rabbit antibody (1:25, clone EPR3864, CliniSciences, Nanterre, France) or anti-CD68 mouse antibody (ref # M087629-2, 1:500, Dako).

***Double Immunofluorescence***

Double immunofluorescence was performed using isolated extracellular vesicles (µV+) from HUVEC stimulated with VEGF (50 ng/mL, 24 h). A medium free of extracellular vesicles (µV-) was used as the control. The two phases were labeled with rabbit anti-FABP4 antibody (ref # ab13979, 1:2000, Abcam, Cambridge, UK), added to HepG2 cells and incubated overnight. β-catenin co-labeling was performed (ref # ab22656, 1:1000, Abcam, Cambridge, UK) for 1 h. Two secondary antibodies were used: Alexa Fluor®488 conjugated goat anti-rabbit IgG (ref # A-11034, 1:1000, Thermo Fisher Scientific®, Waltham, MA, US) and Cyanine 5 conjugated goat anti-mouse IgG (ref # ab6563) (1:1000, Thermo Fisher Scientific®). Counter coloration with DAPI (1:1000; Thermo Fisher Scientific®, Waltham, MA, US) was performed and fluorescent images were obtained on a Leica confocal SP8 (oil objective, x400).

***mRNA quantification and Gene Expression Profiling Analysis***

Total RNA was extracted from HUVEC (basal condition or in presence of Glucose, Insulin, VEGFA or metformin) with an RNAeasy kit (Qiagen) from 3 independent experiments. Real-time quantitative PCR was performed using the Lightcycler 480 system (Roche, Meylan, France). *FABP4* mRNA levels were normalized according to the level of *TBP* RNA control gene.

Total RNA was purified from HepG2 and HuH7 cells incubated or not with eFABP4 (100 ng/mL for 24 h) with an RNAeasy kit (Qiagen) from 4 and 3 independent experiments, respectively. After validation of the RNA quality with Bioanalyzer 2100 (using the Agilent RNA6000 nano chip kit), 80 ng of total RNA was reverse transcribed with the Genechip WT plus Reagent kit (Affymetrix). The resulting double strand cDNAs were used for *in vitro* transcription with T7 RNA pol. After purification, 15 μg of cRNA was used for reverse transcription with random primers. The cDNA obtained was then purified and fragmented. After control of fragmentation using a Bioanalyzer 2100, cDNA was end labelled with biotin using Terminal Transferase (using the WT terminal labelling kit of Affymetrix). cDNA was then hybridized to GeneChip® Human Transcriptome Analysis 2.0 (Affymetrix) at 45°C for 17 h. Chips were washed on the fluidic station FS450 following specific protocols (Affymetrix) and scanned using the GCS3000 7G. The image was then analyzed with Expression Console software (Affymetrix) to obtain raw data (CEL files) and metrics for Quality Controls. Data were normalized using the Expression Console software (Affymetrix) and analyzed with the gene set enrichment analysis (GSEA) software.

***In vivo imaging***

Tumor progression was evaluated using a 7 Tesla MRI system (BioSpec, Bruker BioSpin MRI Gmbh, Ettlingen, Germany) twice, firstly, 2 weeks after tumor cell injection to confirm the presence of a normally growing tumor before beginning treatment, and then 1 and 2 weeks later to evaluate treatment efficiency. Each animal was anesthetized using 1.5% isoflurane at 0.5 L/min oxygen/air. Morphological T2-weighted images were acquired throughout the liver using a RARE (rapid acquisition with relaxation enhancement) sequence with TE/TR of 40/5000 ms, an in-plane resolution of 156 µm, a slice thickness of 1 mm and respiratory gating for a total acquisition time of 10-15 minutes. Using Paravision, tumors were delineated on the T2-weighted images and their total volumes were determined. The tumor growth was assumed to be exponential: the tumor doubling time (days) was calculated as: $TD= \frac{\left( t_{2}-t_{1} \right)ln(2)}{\ln\left( V_{2} \right)-ln(V_{1})}$, where t_1_ and t_2_ are the days where MRI was conducted, and V_1_ and V_2_ the corresponding tumor volumes (mm^3^).

**Legend Supplementary Figures**

**Supplementary Fig. S1. mRNA *FABP4* is regulated in endothelial cells (HUVEC)**. (**A**) FABP4 mRNA expression upon stimulation with glucose (5 and 25 mM). (**B**) FABP4 mRNA expression upon stimulation with insulin (10 and 20 nM). (**C**) FABP4 mRNA expression upon stimulation with VEGFA (10, 25 and 50 ng/ml) for 4 or 24 h. (**D**) FABP4 mRNA expression upon stimulation with VEGFA (50 ng/ml) in presence of metformin (10 μM) for 24 h. The results are expressed as the mean + SD from 3 independent experiments.

**Supplementary Fig. S2. TNFα and not IL6 upregulates FABP4 in HUVEC.** (**A**) FABP4 expression upon stimulation with TNFα (10 and 20 mg/ml). (**B**) FABP4 expression upon stimulation with IL6 (10 and 20 mg/ml). The results are expressed as the mean ± SD from 3 independent experiments.

**Supplementary Fig. S3. FABP4, not expressed in hepatoma cell lines, is upregulated along adipocyte differentiation, hypoxia and presence of free fatty acids. (A)** FABP4 immunoblot performed in hepatoma cell lines (HepG2, HuH7 and SKHep1), in endothelial cells (HUVEC) and in adipocyte 3T3-L1 at three stages of differentiation (pre, intermediate and mature 3T3L1). (**B**) FABP4 expression in HepG2 and HuH7 cell lines subjected to hypoxia for 24 h. (**C**) FABP4 expression in HepG2 and HuH7 cell lines upon stimulation with palmitic acid (200 μM), oleic acid (200 μM) or in combination.
